# Soil degradation regulates the effects of litter decomposition on soil microbial nutrient limitation: Evidence from soil enzymatic activity and stoichiometry

**DOI:** 10.3389/fpls.2022.1090954

**Published:** 2023-01-06

**Authors:** Jianan Li, Ximei Niu, Ping Wang, Jingjing Yang, Jinwen Liu, Donghui Wu, Pingting Guan

**Affiliations:** ^1^ State Environmental Protection Key Laboratory of Wetland Ecology and Vegetation Restoration, School of Environment, Northeast Normal University, Changchun, China; ^2^ Key Laboratory of Vegetation Ecology, Ministry of Education, Northeast Normal University, Changchun, China; ^3^ Jilin Academy of Agricultural Sciences, Jilin, China; ^4^ Key Laboratory of Wetland Ecology and Environment, State Key Laboratory of Black Soils Conservation and Utilization, Northeast Institute of Geography and Agroecology, Chinese Academy of Sciences, Changchun, China

**Keywords:** litter decomposition, grassland degradation, soil enzymes, ecological stoichiometry, nutrient cycling

## Abstract

Soil microorganisms could obtain energy and nutrients during litter decomposition with the help of soil extracellular enzymes. The litter types were among the most critical factors that affect soil extracellular enzyme activities. However, how litter types modulate the soil extracellular enzyme activity with grassland gradation is unclear. Here, we conducted a 240-day experiment of two different types of litter decomposition on soil extracellular enzyme activity and stoichiometry in different degraded grasslands. We found that C-acquiring enzyme activity and the enzyme stoichiometry of C/N were higher in *Chloris virgata* litter than in *Leymus chinensis* litter at lightly degraded level and C-acquiring enzyme activity in *C. virgata* was 16.96% higher than in *L. chinensis*. P-acquiring enzyme activity had the same trend with litter types in moderately and highly degraded levels and it was 20.71% and 30.89% higher in *C. virgata* than that in *L. chinensis*, respectively. The change of the enzyme stoichiometry with litter types was only showed in the enzyme stoichiometry of C/N at lightly degraded level, suggesting that litter types only affected the microbial C limitation in lightly degraded grassland. Almost all soil extracellular enzyme activities and extracellular enzyme stoichiometry, except the enzyme stoichiometry of N/P, decreased with grassland degraded level increasing. All vector angles were less than 45° suggesting that soil microorganisms were limited by N rather than by P during the decomposition process. Enzyme vector analysis revealed that soil microbial communities were co-limited by C and N during litter decomposition. Moreover, based on Random Forest (explaining more than 80%), we found that soil total nitrogen, total carbon, total phosphorus, dissolved organic C, pH and EC were important factors affecting soil enzyme activities by degradation levels. Our results emphasized that degradation levels could modulate the influences of litter types on soil extracellular enzyme activity. Our study enhanced our understanding in resource requirements for microbial communities to litter resources in degraded grassland and helped us to provide new ideas for improving degraded grassland ecosystems.

## 1 Introduction

Litter decomposition plays an important role in nutrient cycling in terrestrial ecosystems (Wu et al., 2021; Zhao et al., 2021; Zhang et al., 2022). Different qualities of litter inputs change the nutrient contents of soil (e.g., C, N, P, etc.), which subsequently leads to changes in soil extracellular enzyme activity (Xu et al., 2013; Zeng et al., 2018). Soil extracellular enzymes are mainly produced by microorganisms and are closely involved in the effectiveness of soil carbon decomposition and nutrient mineralization (Sinsabaugh et al., 2009). The activities of these enzymes are frequently linked to rates of microbial metabolism and mediated microbial nutrient acquisition. Therefore, extracellular enzyme activity (EEA) is commonly interpreted as an efficient indicator of nutrient acquisition by microorganisms (Moorhead et al., 2016). Many studies have shown that, compared with without litter, litter addition can affect the soil extracellular enzyme activity, and generally improve the soil extracellular enzyme activity and relieve microbial nutrient limitation (Weintraub et al., 2013; Kotroczo et al., 2014; Zeng et al., 2021; Wu et al., 2022). At the same time, studies find that litter type is one of the most important factors in shaping soil microbial community structure and enzyme secretion (Urbanova et al., 2015; Sayer et al., 2017; Vivelo and Bhatnagar, 2019), but consistent conclusions are still lacking (Tan et al., 2020; Liu J et al., 2022). Differences in the ecological environment, especially in the soil environment, could alter the role of litter type in influencing EEA. Therefore, the effects of litter types on EEA in degraded grassland still need to deeply explore (Brandt et al., 2010; Chuan et al., 2020).

It has been shown that soil EEA varies during decomposition in response to changes in litter quality and soil condition (Fioretto et al., 2007). Indicators commonly used for litter quality, such as nitrogen (N), phosphorus (P), C/N, and C/P, are correlated with soil EEA (Fang et al., 2007; Gong et al., 2020). Large variations in litter N and P content may lead to changes in N- and P-acquiring enzyme activities during decomposition (Gong et al., 2022), as well as changes in soil extracellular enzyme stoichiometry (EES). Soil extracellular enzyme stoichiometry is the ratio of EEAs related to the acquisition of nutrients, and illustrates the relationship between soil nutrient availability and microbial nutritional demands (Sinsabaugh et al., 2009). Sinsabaugh et al. (2008) suggested that EES could be used as an effective tool to evaluate the biogeochemical equilibrium between metabolic and nutrient requirements of microbial assemblages and the nutrient availabilities of the environment at a global scale. Globally, soil EES is strongly constrained, with a mean C: N: P ratio of enzyme activities near 1:1:1 of C-, N-, and P-acquiring enzymes (Sinsabaugh et al., 2008; Sinsabaugh et al., 2009). Therefore, soil extracellular enzyme stoichiometry is an efficient indicator of microbial nutrition requirements and closely correlates with soil nutrient stoichiometry (Deng L et al., 2019; Cui et al., 2021). When litter with different quality or stoichiometric characteristics decomposes, soil microorganisms adapt to these different input nutrients by regulating the enzyme stoichiometry of C/N, C/P, or N/P (Mooshammer et al., 2014; Liu et al., 2019).

Studies have shown that EEA and EES were regulated by soil pH, and nutrient availability, including stoichiometry of soil (Sinsabaugh et al., 2008; Hewins et al., 2015). Enzymes have long been recognized to have an optimal pH (Wade et al., 2021; Liu M et al., 2022). Soil pH not only affects enzyme activity by directly altering the enzyme conformation (Wade et al., 2021) but also has a significant impact on microbial activity, as soil pH may lead to changes in cell membrane charge and affect nutrient uptake by microorganisms, thus affecting their survival and reproduction (Rath et al., 2019). In addition, soil nutrients have an important effect on enzyme activity. It was found that both the soil nutrient content of grassland (Fanin et al., 2016), and soil nutrient stoichiometry (Cui et al., 2019) can lead to changes in soil EES. In particular, changes in soil nutrient stoichiometry may result in soil nutrient imbalance that compromises microbial activity (Chen et al., 2022), which subsequently alters soil extracellular enzymes and stoichiometry. Thus, the effect of litter type on soil EEA and EES may be modulated by these soil factors when one or some of the soil factors are largely different, which interferes with our understanding of the relationship between litter input and soil EEA and EES.

Grassland ecosystems play a crucial role in the nutrient cycling of terrestrial ecosystems on Earth (Bengtsson et al., 2019; Wu et al., 2021). However, more than half of the world’s grassland has been severely degraded due to intensive anthropogenic and global climate change, not only altering soil physicochemical properties (i.e., salinity, moisture, and nutrient availability) (Wu et al., 2021), but also negatively affecting ecosystem productivity, stability, and sustainability (Bai et al., 2012; Zhou et al., 2019; Yang et al., 2021). Continuous input of litter in degraded grasslands can play an important role in regulating soil nutrients and maintaining productivity (Gong et al., 2022). To investigate the effects of litter types on soil enzyme activity and nutrient limitation in degraded grasslands, we conducted a 240-day decomposition experiment. This study aimed to investigate (1) the effects of different soil degradation levels on soil extracellular enzyme activity and stoichiometry, and (2) how the effect of different litter types on soil extracellular enzyme activity and stoichiometry was regulated by soil degradation levels.

## 2 Materials and methods

### 2.1 Study site

The study was conducted in the Grassland Ecological Research Station of Northeast Normal University, Jilin province, China (44°45’N, 123°45’E). This region is characterized by a semi-arid continental monsoon, with cold dry winters and warm rainy summers. The mean average temperature is 4.6- 6.4°C, and the mean annual precipitation is 282 - 400 mm, with most falling between June and August (Wang et al., 2017). The soil type is saline-alkaline meadow soil with a high degree of soil heterogeneity. The soil pH in the study area ranges from 8.5 to 10.0 (Li et al., 2015). The land type is mainly grassland. Based on soil electrical conductivity (EC), pH, soil properties, the plant covers and the actual conditions of study area, there are three levels of degradation in this region: lightly, moderately, and highly degraded grassland (Pan et al., 2013; Wu et al., 2021; Yang et al., 2022). The dominant species in lightly degraded grassland is *Leymus chinensis*, while *L.chinensis*, *Chloris virgata* and are dominant in moderately degraded grassland, *C. virgata*, *Suaeda corniculata*, *Puccinellia distans* are dominant in highly degraded grassland (Wang L et al., 2019; Yang et al., 2021).

### 2.2 Decomposition experiment

Soil samples were collected from the three levels of degraded grasslands (i.e. three soil treatments) on October 13th, 2019. Four plots (25 m × 25 m for each) were randomly selected as replicate soil samples on each level of degraded grassland (12 plots in total). After the removal of surface impurities, 20 cores were randomly collected in each plot from a depth of 0-10 cm using a soil auger (5 cm diameter) and mixed thoroughly. There was about 6 kg of soil in total was collected as one representative soil sample from each plot. We collected the above-ground material of *L. chinensis* from the *L. chinensis*-dominated communities and *C. virgata* from the *C. virgata*-dominated communities, respectively, as litter for the subsequent decomposition experiment. All litter and soil samples were transported to the laboratory and stored at 4°C. The initial carbon, nitrogen, and phosphorus contents and elemental ratios of soil and litter samples, as well as the electrical conductivity and pH of the soil, were measured and listed in **Tables S1**, **S2**.

Two litter treatments were set up for this study: *L. chinensis* litter and *C. virgata* litter. After air dried, the collected litter of *L. chinensis* and *C. virgata* were cut into pieces approximately 1 cm long. There was 3.00 g of each litter was put into one plastic litter bag (1 mm mesh). The soil samples were passed through an 8-mm sieve to remove stones and roots. A total of 400 g of fresh soil was weighed in a PVC tube (9 cm inner diameter and 15 cm high). There were 24 PVC tubes in total (3 degraded soil × 2 litter types × 4 replicates). All PVC tubes were pre-incubated in an incubator under standardized conditions (25°C; soil humidity 60% of saturation) for 7 days to balance water and nutrients (Maggiotto et al., 2019). A litter bag was then placed on the soil surface of each PVC tube according to the experimental design. The experiment began on October 21, 2019. All the tubes were weighed and rehydrated every 2 days to maintain a 60% water-holding capacity during the experiment period (Creamer et al., 2015). PVC tubes were placed in an incubator under standardized conditions with the same condition as pre-incubated.

After 240 days, on June 27, 2020, soil samples and litter bags were collected from PVC tubes. Litters were gently sieved through a 2 mm sieve to remove any adhering soil particles and then oven-dried at 60 °C for 48 h to reach a constant weight. Soil samples were stored in plastic bags and maintained at 4°C for further measurements.

### 2.3 Measurements of litter and soil physicochemical properties

Soil pH and electrical conductivity (EC) were measured using a pH meter and conductivity meter in a 1:5 (w/v) solution of soil–deionized water. Soil moisture (SM) was determined gravimetrically by weight method after drying the samples at 105°C for 24 h. Soil inorganic nitrogen ( ${\text{NO}}_{3}^{-}$ and ${\text{NH}}_{4}^{+}$ ) was extracted with 2 mol KCl and determined using an automatic elemental analyzer (SmartChem 200). Dissolved organic C was extracted with deionized water and filtered through a 0.45μm membrane, and then quantified using a total C analyzer (TOC VCPH, Shimadzu) (Wu et al., 2015). The contents of total nitrogen and total carbon in the litter (LTN and LTC) and soil samples (STN and STC) were determined by an automatic elemental analyzer (Flash EA 1112 elemental, Italy). The contents of total phosphorus in the litter (LTP) and soil (STP) samples were detected by a continuous flow analyzer (Skalar, the Netherlands).

### 2.4 Soil enzyme assays measurement and enzyme stoichiometry determination

The activities of soil extracellular enzymes, including C- acquiring enzyme (β-1,4-glucosidase (BG) and cellobiose (CB)), N- acquiring enzyme (β-1,4-N-acetyl glucosaminidase (NAG) and 1-leucine aminopeptidase (LAP)), and P- acquiring enzyme (alkaline phosphatase, (AP)), were assayed using a microplate protocol (Sinsabaugh et al., 2008). The enzyme activity was expressed as nmol g^−1^soil h^−1^.

Two methods were used to assess microbial resource limitation by enzyme stoichiometry determination: 1) the enzyme stoichiometry was the radio of C-, N- and P-acquiring enzyme activity, which enabled the estimation of potential C, N and P limitation in soils (Hill et al., 2012), specifically the enzyme stoichiometry of C/N, N/P, and C/P (Waring et al., 2014; Moorhead et al., 2016); 2) vector analysis of enzymatic stoichiometry (Moorhead et al., 2013). Vector length (unitless) and vector angle (degree) were calculated as follows:


$$Vector\;length=\sqrt{{\left(\frac{\ln C}{\ln N}\;\right)}^{2}+{\left(\frac{\ln C}{\ln P}\right)}^{2}}$$

$$Vector\;angle=Degrees\;\left(ATAN2\left(\left(\frac{\ln C}{\ln P}\right),\;\left(\frac{\ln C}{\ln N}\right)\right)\right)$$

(C-acquiring enzyme activity; N, N-acquiring enzyme activity; P, P-acquiring enzyme activity.)

A longer vector length indicates greater C limitation, and the vector angles of < 45° and > 45° indicate N and P limitation, respectively (Moorhead et al., 2013).

### 2.5 Data analysis

All analyses and figures were carried out with R software version 4.0.3 (R Development Core Team, 2022). The effects of litter types and degraded levels on soil and litter physicochemical properties, extracellular enzyme activity, and extracellular enzyme stoichiometry were determined using two-way ANOVA with litter types, degraded levels, and their interactions as fixed factors. Duncan’s multiple comparisons were applied as a *post hoc* analysis to test for significant differences among litter types and degraded levels. Principal coordinates analysis (PCoA) was used to describe differences in soil extracellular enzyme activity and stoichiometry among three degraded levels using the Vegan package. Random Forest was performed to create models that describe the relationship between soil extracellular enzyme activity, soil properties, and litter properties using the rfPermute and A3 package. Differences at P< 0.05 were considered significant.

## 3 Results

### 3.1 Soil extracellular enzymes and stoichiometry after decomposition

After 240-day incubation, litter types showed significant effects on C-, P-acquiring enzyme activity and the enzyme stoichiometry of C/N (P< 0.05, **Table 1**). All soil EEA and EES were significantly affected by degraded levels (P< 0.01). The interaction of degraded levels and litter types only showed significant effects on C-acquiring enzyme activity (P< 0.05). Litter types did not affect vector length and angle. Vector length and angle were significantly affected by degraded levels (P< 0.01). The interaction effect of degraded levels and litter types was not found on vector length and angle (P > 0.05, **Table 1**).

**Table 1:** Results of a two-way ANOVA for degraded levels and litter types.

treatment	C- enzyme	N- enzyme	P- enzyme	C/N	C/P	N/P	Vector length	Vector angle
L	0.028*	0.723	0.002**	0.027*	0.452	0.052	0.484	0.052
D	P< 0.01**	P< 0.01**	P< 0.01**	P< 0.01**	P< 0.01**	P< 0.01**	P< 0.01**	P< 0.01**
L × D	0.034*	0.918	0.843	0.369	0.208	0.896	0.45	0.803

L, litter types; D, degraded levels; L x D, the interaction of degraded levels and litter types. C- enzyme, C-acquiring enzyme activity; N- enzyme, N-acquiring enzyme activity; P- enzyme, P-acquiring enzyme activity; C/N, the enzyme stoichiometry of C/N; C/P, the enzyme stoichiometry of C/P; N/P, the enzyme stoichiometry of N/P. *P< 0.05; **P< 0.01.

**Figure 1:**
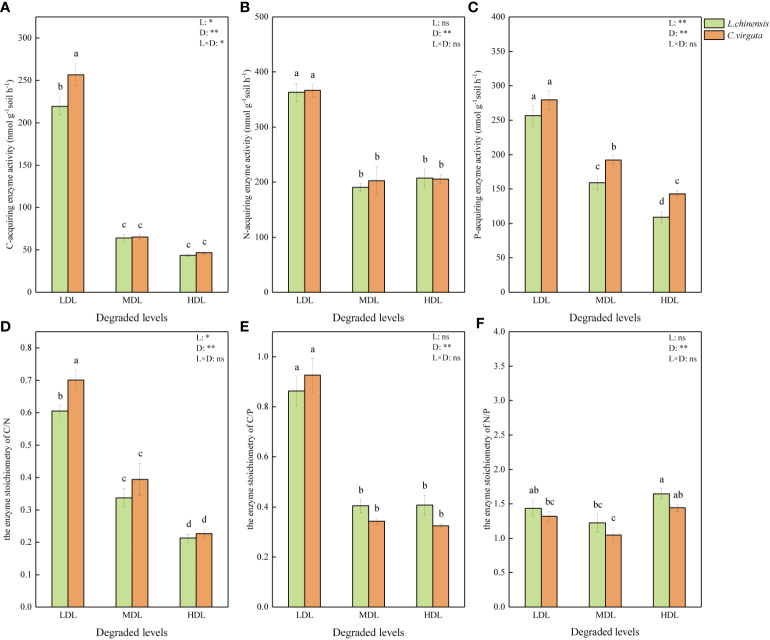
Soil extracellular enzyme activity (nmol g^−1^soil h^−1^) and stoichiometry under influence of different litter types at different degraded levels. (LDL, lightly degraded level; MDL, moderately degraded level; HDL, highly degraded level; *L. chinensis*, *Leymus chinensis*; *C. virgata*, *Chloris virgata*. **(A)** C-acquiring enzyme activity; **(B)** N-acquiring enzyme activity; **(C)** P-acquiring enzyme activity; **(D)** the enzyme stoichiometry of C/N; **(E)** the enzyme stoichiometry of C/P; **(F)** the enzyme stoichiometry of N/P. Different letters represent significant differences among means at P < 0.05).

All soil EEA and EES were highest at lightly degraded level, except the enzyme stoichiometry of N/P (P< 0.05, **Figure 1**). The C-acquiring enzyme activity was higher in C. virgata treatment than in L. chinensis treatment at lightly degraded level, and the enzyme stoichiometry of C/N had the same trend (P< 0.05). The P-acquiring enzyme activity was higher in C. virgata treatment than L. chinensis treatment in moderately and highly degraded levels (P< 0.05). But there was no difference between N-acquiring enzyme activity and the enzyme stoichiometry of C/P and N/P among litter types in all three degraded levels (P > 0.05, **Figure 1**).

**Figure 2:**
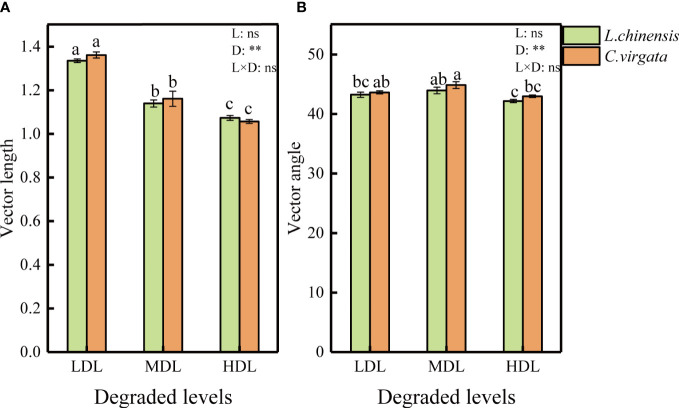
Vector length and angle under influence of different litter types at different degraded levels. (LDL, lightly degraded level; MDL, moderately degraded level; HDL, highly degraded level; *L. chinensis*, *Leymus chinensis*; *C. virgata*, *Chloris virgata*. **(A)** Vector length; **(B)** Vector angle. Different letters represent significant differences among means at P < 0.05).

Vector length had the highest value at the lightly degraded level but the angle at the moderately degraded level (P< 0.01, **Figure 2**). Vector length and angles had no difference between litter types. All vector angles were < 45°, suggesting that soil microorganisms were more limited by N than by P during the decomposition process.

**Figure 3:**
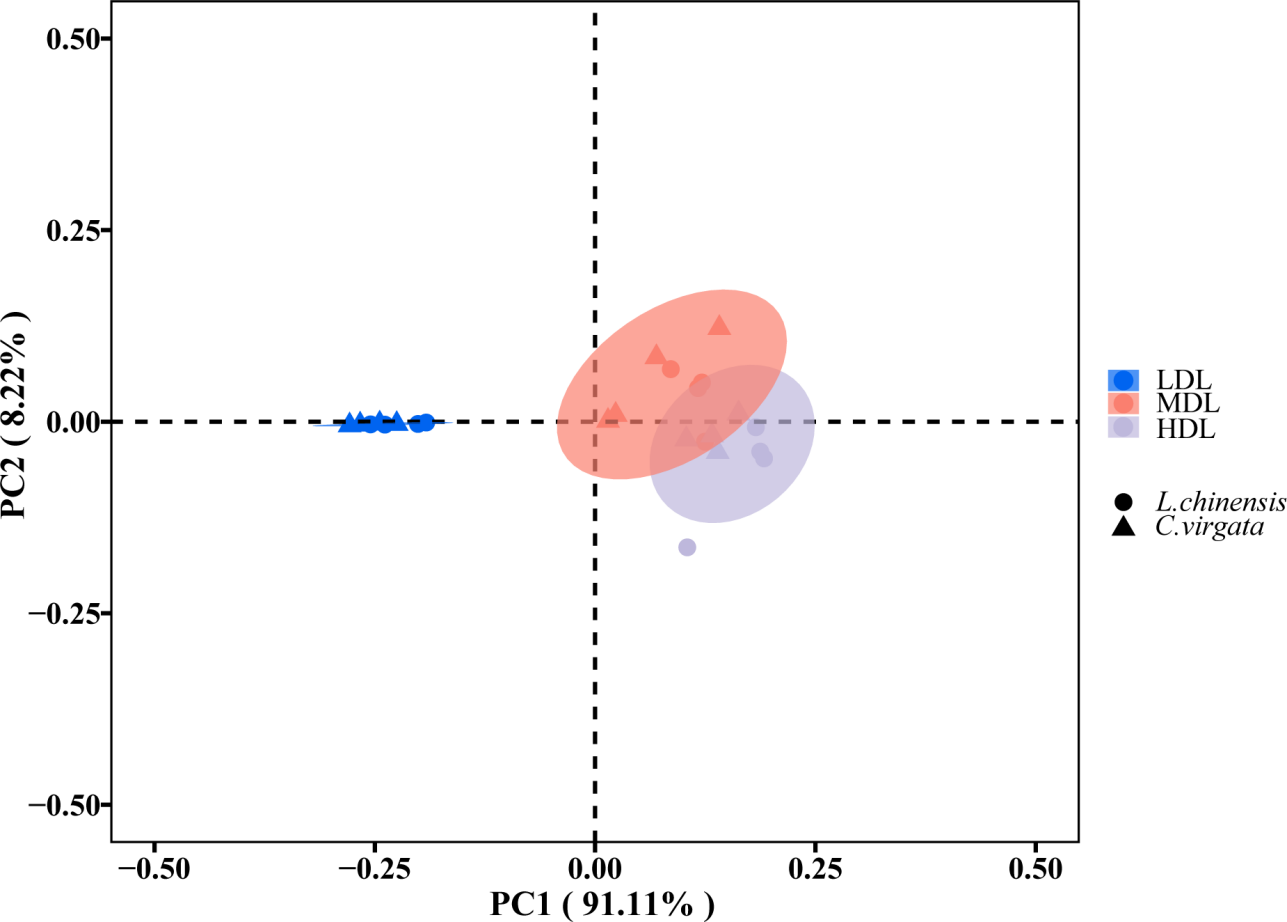
Principal coordinates analysis describing differences in soil extracellular enzyme activity (nmol g^−1^soil h^−1^) and stoichiometry among three degraded levels. (LDL, lightly degraded level; MDL, moderately degraded level; HDL, highly degraded level; *L. chinensis*, *Leymus chinensis*; *C. virgata*, *Chloris virgata*).

The relationships of EEA and EES between different degraded levels and different litter types were expressed by PCoA (F = 105.85, P< 0.01) (**Figure 3**). The two litter types were clustered together at the lightly degraded level. At the moderately and highly degraded levels, litter types clustered together at each degraded level, but not as pronounced as at the lightly degraded level (**Figure 3**).

### 3.2 Influence of soil and litter properties on soil extracellular enzyme activities

**Figure 4:**
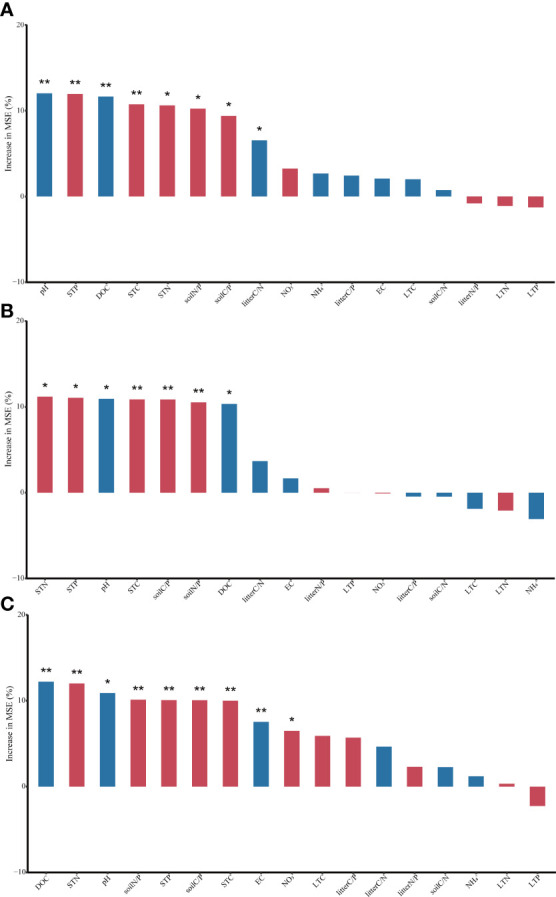
Soil enzyme activity (nmol g^−1^soil h^−1^) was related to soil properties and litter properties using Random Forest. (STC, soil total carbon; STN, soil total nitrogen; STP, soil total phosphorus; LTC, litter total carbon; LTN, litter total nitrogen; LTP, litter total phosphorus. **(A)** C-acquiring enzyme activity; **(B)** N-acquiring enzyme activity; **(C)** P-acquiring enzyme activity. Red columns, a positive correlation; blue columns, a negative correlation. *P < 0.05; **P < 0.01).

The results of the random forest analysis showed that both C-, N- and P-acquiring enzyme activities (P< 0.05) were affected by pH, DOC, STC, STN, STP, soil C/P and soil N/P (**Figure 4**). Additionally, C-acquiring enzyme activity was also influenced by litter C/P, and P-acquiring enzyme activity was also influenced by EC and ${\text{NO}}_{3}^{-}$ .

## 4 Discussion

### 4.1 Effect of soil degradation on soil extracellular enzyme activity and stoichiometry

After 240-day of incubation, all the soil EEA we measured decreased with increasing levels of soil degradation (**Figure 1**). As soil C-, N- and P-acquiring enzyme activities were all significantly and positively correlated with soil nutrient contents (**Figure 4**), when STC, STN, and STP contents decreased with increasing levels of soil degradation, soil C-, N- and P-acquiring enzyme activities also decreased. Our study and previous studies have shown that the low nutrient content of degraded grassland soils inhibited the activity of soil extracellular enzymes (Gao et al., 2014; Wang Y et al., 2019).

In addition, the activities of C-, N- and P-acquiring enzymes all decreased significantly with increasing of soil pH (**Figure 4**). Degraded grasslands, following a reduction in vegetation cover, allow salt-rich groundwater in deep soils to accumulate on the soil surface with surface evaporation, increasing surface soil pH (Li and Zhou, 2018). Soil pH affected enzyme kinetics by altering substrate binding and stability (Puissant et al., 2019), and the inhibitory effect of elevated soil pH on soil EEA was progressively enhanced as soil degradation increased. Soil P-acquiring enzyme activity was also influenced by EC (**Figure 4C**). Microorganisms associated with phosphorus decomposition might be relatively sensitive to increased soil salinity concentrations, resulting in a disruption of the internal and external osmotic pressure equilibrium, which reduced their activities (Xi et al., 2014). Therefore, soil P-acquiring enzyme activity decreased with increased soil degradation (**Figure 1C**).

Changes in soil properties in degraded grassland not only significantly affected the activity of soil extracellular enzymes, but also significantly altered the stoichiometry of soil extracellular enzymes (**Figures 1**, **2**). In all three degraded soils, the extracellular enzyme vector angles were less than 45°, indicating that microorganisms in all degraded soils were N-limited, and the N limitation of microorganisms was significantly enhanced in heavily degraded soils (**Figure 2B**). Severely degraded grasslands were generally in a nutrient- deficiency state, especially lacking in N-related nutrients (Luo et al., 2022). The total and available nitrogen contents of highly degraded soils (**Tables S1**, **S3**) were significantly lower than those of the other two degraded soils. In such low-nitrogen soils, microorganisms can only dissolve and utilize soil N by synthesizing a high percentage of N-acquiring enzymes to meet nutrient requirements. Correspondingly, the proportion of extracellular enzyme content of other types subsequently decreased, showing enzyme stoichiometry trends of lower C/N and higher N/P, respectively (**Figures 1D, F**).

In contrast to N limitation, the enzyme stoichiometry of C/N, C/P, and vector length decreased with increasing levels of soil degradation (**Figures 1D, E** and **Figure 2A**), indicating that the C limitation of soil microorganisms decreased with increasing soil degradation. The increasing of DOC content with increasing degradation levels might explain the microbial C limitation. As for a soluble component, DOC was easily affected by leaching, which accelerated the decomposition and released of soluble substances (Nakhavali et al., 2021). DOC was active carbon that was easy for microorganisms to absorb and use. Degradation had a contribution to the input of carbon from litter (Maxwell et al., 2019). This might be one of the reasons for the decrease in microbial C limitation in moderately and highly degraded levels. In addition, the coupling between C and N cycling might also affect C limitation. The C cycle process and its turnover rate were directly related to the N availability (Marklein and Houlton, 2012). The litter deposition released a large amount of C and N which affected N mineralization–immobilization turnover and soil C sequestration (Soussana and Lemaire, 2014). Accompanied by increasing N limitation, low N availability could increase litter decomposition, promoting labile C was utilized and then alleviated microbial C limitation (Craine et al., 2007). Accordingly, microbial demand for soil carbon and nitrogen was interdependent. Our results showed that both microbial carbon limitation and nitrogen limitation were affected by the level of soil degradation, reflecting that changes in soil properties can significantly alter microbial demand for different nutrients and subsequently affect soil extracellular enzyme activity and enzyme stoichiometry.

### 4.2 The effects of litter types on soil extracellular enzyme activities and enzyme stoichiometry are related to soil degradation level

The chemical properties of plant litter affected soil extracellular enzyme activities and regulated ecological enzyme stoichiometry (Zeng et al., 2021). Our results showed that the effect of litter types on EEA and EES varied with the degree of degradation, i.e. litter types significantly affected C-acquiring enzyme activity and enzyme stoichiometry of C/N in lightly degraded soil (**Table 1**, **Figures 1A, D**), while it significantly affected P-acquiring enzyme activity in moderately and highly degraded soils, (**Table 1** and **Figure 1C**). In addition, no effect of litter types on N-acquiring enzyme activity was found in this study (**Table 1** and **Figure 1B**).

In lightly degraded soil, both C-acquiring enzyme activity and enzyme stoichiometry C/N were lower under *L. chinensis* litter than *C. virgata* litter (**Figures 1A, D**). There were several possible reasons for this result. On the one hand, the initial C content of *L. chinensis* litter was significantly higher than that of *C. virgata* litter (**Table S2**), which could provide more carbon elements for microorganisms and thus reduce the secretion of related extracellular enzymes. On the other hand, “home-field advantage” (HFA) may also be at play. The HFA suggests that plant litter decomposes faster in its natural habitat than in other environments because soil microorganism communities have adapted to the local microenvironments and chemical compositions of plant litter (Ayres et al., 2009; Wang et al., 2013; Chomel et al., 2015; Palozzi and Lindo, 2018). *L. chinensis* is the dominant species in lightly degraded grassland (Wang L et al., 2019), and *C. virgata* is the dominant species in highly degraded grassland (Cao et al., 2019; Yang et al., 2021). Therefore, microorganisms in lightly degraded soils exhibited a lower C-acquiring enzyme activity to decompose and utilize C from native *L. chinensis* litter relative to nonnative *C. virgata* treatment.

The effect of litter types on C-acquiring enzyme activity disappeared in moderately and highly degraded soils (**Figure 1A**). The home-field advantage of native *C. virgata* litter by soil microorganisms in moderately and highly degraded soils may have diminished the effect of initial litter carbon content differences on C-acquiring enzyme activity. In addition, the C limitation of soil microorganisms gradually decreased and the N limitation increased with increasing soil degradation (**Figures 1**, **2**). Since extracellular enzyme production requires metabolic energy consumption (Mooshammer et al., 2014; Zhou et al., 2020), microorganisms substantially reduced the C-acquiring enzyme secretion (**Figure 1**), to allocate energy to produce more N-acquiring enzymes. Thus, the decrease in C-acquiring enzyme activity due to soil degradation weakened the effect of different litter types on C-acquiring enzyme activity.

On the contrary, litter type did not affect P-acquiring enzyme activity in lightly degraded soils, but significantly affected P-acquiring enzyme activity in moderately and highly degraded soils, i.e. lower P-acquiring enzyme activity in *L. chinensis* treatment than that in *C. virgata* treatment (**Figure 1C**). There was no difference in the initial P content between *L. chinensis* and *C. virgata* litter (**Table S3**). Therefore, the litter P input may not be the main factor that caused significant differences in P-acquiring enzyme activity between litter treatments. In moderately and highly degraded soils, the variation of soil available C and N nutrients between *L. chinensis* litter and *C. virgata* litter might lead to a change in P-acquiring enzyme activity (**Figure 4C**). Similar results have been shown in previous studies (Ren et al., 2016; Pan et al., 2018; Deng J et al., 2019). Although P-acquiring enzyme activity differed between litter types, enzyme stoichiometry of C/P, N/P and vector angle did not change, indicating that *L. chinensis* litter and *C. virgata* litter were not sufficient to alter the nutrient demands of microorganisms for P.

The N-acquiring enzyme activity was not affected by litter types in this study (**Figure 1B** and **Table 1**), probably because the soil was under N limitation. Grassland soils were usually at low levels of N and lack both fertilizer inputs of agricultural systems and abundant N-fixing bacteria of forest systems (Fanin et al., 2016). Although the initial litter N content of *L. chinensis* litter was significantly higher than that of *C. virgata* litter (**Table S2**), differences in litter N input did not change N-acquiring enzyme activity and the original nutrient limitation of grassland soil. Moderately and highly degraded grassland are nutrient-poor (Luo et al., 2022), and soil extracellular enzyme activities were weakened by litter and were more susceptible to degraded soils.

## 5 Conclusions

We used soil extracellular enzyme activity and stoichiometry to investigate the changes of soil extracellular enzyme activity and nutrient limitation with different litter types in degraded grassland. Litter types could influence soil extracellular enzyme activities. Compared with *L. chinensis*, *C. virgata* input stimulated the microorganisms to produce more soil extracellular enzymes to obtain nutrients. This effect would vary with different degraded levels, which reflected the regulation of degradation level on litter decomposition. In lightly degraded grasslands with relatively sufficient nutrients, soil extracellular enzyme activities were more susceptible to differences between litter types. While in moderately and highly degraded grassland with poor nutrients, the soil properties would become the dominant factor on extracellular enzyme activities rather than litter types. Therefore, the effect of litter types on soil enzyme activity was modulated by different degraded levels. In degraded levels, soil physicochemical properties had greater effects on soil extracellular enzyme activity. The degraded levels determined soil extracellular enzyme activity and microbial nutrient limitation, whereas litter types only partly altered soil enzyme activities. We emphasized the importance of litter in degraded grasslands by using soil extracellular enzymes and stoichiometry and highlighted the strong influence of soil physicochemical properties on soil extracellular enzyme activity and stoichiometry.

## Data availability statement

The original contributions presented in the study are included in the article/**Supplementary Material**. Further inquiries can be directed to the corresponding author.

## Author contributions

PG and PW contributed to the study’s conception and design. JL, XN, JWL and JY performed the material preparation and data collection. JL and JY performed the data analyses. JL wrote the manuscript. PG, PW and DW revised the manuscript. All authors commented on previous versions of the manuscript, read, and approved the final manuscript.

## Supplementary material

Supplementary Table 4 Litter properties and ratios after 240-days decomposition.
