# Supplementary material for: Soil degradation regulates the effects of litter decomposition on soil microbial nutrient limitation: Evidence from soil enzymatic activity and stoichiometry
Source: Front Plant Sci. 2023 Jan 6;13:1090954. doi: 10.3389/fpls.2022.1090954 (PMC9853160; doi:10.3389/fpls.2022.1090954)
Supplement: Supplementary Table 4 — Litter properties and ratios after 240-days decomposition. [file Table_1.docx]

| Degraded levels | EC(μs/cm) | pH | NH_4_^+^ (mg/kg) | NO_3_^-^ (mg/kg) | DOC (g/kg) | STC (g/kg) | STN (g/kg) | STP (g/kg) | Soil C/N | Soil C/P | Soil N/P |
| --- | --- | --- | --- | --- | --- | --- | --- | --- | --- | --- | --- |
| LDL | 85.38±1.36c | 8.32±0.08c | 0.10±0.03 | 0.44±0.02c | 0.70±0.03b | 15.49±0.14a | 2.22±0.00a | 0.30±0.01 | 6.99±0.06a | 51.61±1.96a | 7.39±0.31 |
| MDL | 223.25±9.26b | 9.81±0.02b | 0.22±0.09 | 1.34±0.06b | 1.32±0.42ab | 11.47±0.47b | 1.97±0.06b | 0.28±0.01 | 5.80±0.09b | 41.29±0.62b | 7.12±0.08 |
| HDL | 314.50±20.62a | 10.04±0.02a | 0.10±0.04 | 1.56±0.06a | 1.92±0.27a | 10.60±0.27b | 1.88±0.05b | 0.26±0.00 | 5.66±0.24b | 40.38±1.22b | 7.15±0.19 |

**Table S1** The soil properties in study sites before decomposition.

LDL, lightly degraded level; MDL, moderately degraded level; HDL, highly degraded level; STC, soil total carbon; STN, soil total Nitrogen; STP, soil total phosphorus. Different lowercase letters indicate significant differences with degraded levels (P < 0.05).

**Table S2** The litter properties in study sites before decomposition.

| Litter types | LTC (g/kg) | LTN (g/kg) | LTP (g/kg) | Litter C/N | Litter C/P | Litter N/P |
| --- | --- | --- | --- | --- | --- | --- |
| *L. chinensis* | 461.10±1.20a | 18.16±0.23a | 3.60±0.08 | 25.40±0.26 | 128.32±2.63a | 5.05±0.10a |
| *C. virgata* | 443.11±2.42b | 14.06±0.75b | 3.89±0.10 | 29.04±2.78 | 114.25±2.81b | 3.68±0.35b |

*L. chinensis*, *Leymus chinensis*; *C. virgata*, *Chloris virgata*; LTC, litter total carbon; LTN, litter total Nitrogen; LTP, litter total phosphorus. Different lowercase letters indicate significant differences with degraded level or litter types (*P* < 0.05).

**Table S3** Soil properties and ratios after 240-days decomposition.

| Degraded levels | Litter types | EC (μs/cm) | pH | NH_4_^+^ (mg/kg) | NO_3_^-^ (mg/kg) | DOC (g/kg) | STC (g/kg) | STN (g/kg) | STP (g/kg) | Soil C/N | Soil C/P | Soil N/P |
| --- | --- | --- | --- | --- | --- | --- | --- | --- | --- | --- | --- | --- |
| LDL | *L. chinensis* | 177.98±12.44B | 8.19±0.06bC | 0.01±0.00bB | 0.66±0.10bA | 0.35±0.03B | 16.55±0.54A | 1.92±0.05A | 0.31±0.00bA | 8.64±0.12A | 53.16±2.11A | 6.15±0.18A |
|  | *C. virgata* | 177.40±18.90B | 8.47±0.05aC | 0.07±0.01aB | 1.53±0.24aA | 0.35±0.04B | 16.34±0.57A | 1.92±0.06A | 0.33±0.00aA | 8.53±0.03A | 49.52±1.57A | 5.80±0.16A |
| MDL | *L. chinensis* | 149.08±2.30B | 9.56±0.04B | 0.03±0.01bAB | 0.90±0.15A | 0.85±0.19bA | 12.06±0.20B | 1.36±0.02B | 0.29±0.01B | 8.90±0.05A | 42.04±1.61B | 4.72±0.18B |
|  | *C. virgata* | 157.80±32.88B | 9.52±0.17B | 0.09±0.00aAB | 1.12±0.17A | 2.03±0.34aA | 11.44±0.12B | 1.32±0.05B | 0.29±0.00B | 8.70±0.32A | 39.50±0.88B | 4.56±0.22B |
| HDL | *L. chinensis* | 237.18±21.52A | 9.88±0.11bA | 0.07±0.01A | 0.36±0.08B | 0.60±0.04bA | 10.26±0.34C | 1.14±0.03C | 0.28±0.01B | 9.00±0.07A | 36.59±1.60C | 4.07±1.17C |
|  | *C. virgata* | 305.75±25.09A | 10.16±0.02aA | 0.09±0.01A | 0.41±0.10B | 1.19±0.17aA | 10.08±0.24C | 1.16±0.01C | 0.29±0.00B | 8.67±0.17A | 34.84±1.00C | 4.02±0.06C |
| L |  | P < 0.01 | P < 0.01 | P < 0.01 | P < 0.01 | P < 0.01 | P < 0.01 | P < 0.01 | P < 0.01 | ns | P < 0.01 | P < 0.01 |
| D |  | P < 0.01 | P < 0.01 | P < 0.01 | P < 0.01 | P < 0.01 | P < 0.01 | P < 0.01 | P < 0.05 | ns | P < 0.01 | P < 0.01 |
| L × D |  | ns | ns | P < 0.01 | ns | ns | ns | ns | ns | ns | ns | ns |

Values are the means (± standard errors) of four replicate soil cores. L, litter types; D, degraded levels; L × D, the interaction of degraded levels and litter types; LDL, lightly degraded level; MDL, moderately degraded level; HDL, highly degraded level; *L. chinensis*, *Leymus chinensis*; *C. virgata*, *Chloris virgata*; STC, soil total carbon; STN, soil total Nitrogen; STP, soil total phosphorus. Different capital letters indicate significant differences with degraded levels. Different lowercase letters indicate significant differences with litter types in the same degraded level (*P* < 0.05).

**Table S4** Litter properties and ratios after 240-days decomposition.

| Degraded levels | Litter types | LTC (g/kg) | LTN (g/kg) | LTP (g/kg) | Litter C/N | Litter C/P | Litter N/P |
| --- | --- | --- | --- | --- | --- | --- | --- |
| LDL | *L. chinensis* | 123.44±8.15A | 8.18±0.77A | 3.71±0.19A | 15.61±2.03bB | 33.68±3.32A | 2.21±0.19A |
|  | *C. virgata* | 198.58±46.88A | 9.24±0.73A | 3.91±0.13A | 14.74±1.13bB | 50.34±11.45A | 2.43±0.17A |
| MDL | *L. chinensis* | 171.99±22.64A | 8.73±1.03A | 3.94±0.11A | 19.60±0.73A | 43.80±6.16A | 2.22±0.28AB |
|  | *C. virgata* | 183.92±27.43A | 8.58±1.22A | 3.72±0.15A | 21.48±2.08A | 49.41±7.30A | 2.29±0.25AB |
| HDL | *L. chinensis* | 117.15±16.00bA | 5.46±0.41bB | 3.62±0.05A | 21.36±1.90bA | 32.26±4.01bA | 1.51±0.11bB |
|  | *C. virgata* | 247.28±33.93aA | 8.56±0.79aB | 3.83±0.16A | 26.64±0.86aA | 63.80±6.85aA | 2.22±0.12aB |
| L |  | P < 0.05 | ns | ns | ns | P < 0.05 | ns |
| D |  | ns | P < 0.05 | ns | P < 0.05 | ns | P < 0.01 |
| L × D |  | P < 0.05 | ns | ns | P < 0.05 | ns | ns |

Values are the means (± standard errors) of four replicate litter bags. L, litter types; D, degraded levels; L × D, the interaction of degraded levels and litter types; LDL, lightly degraded level; MDL, moderately degraded level; HDL, highly degraded level; *L. chinensis*, *Leymus chinensis*; *C. virgata*, *Chloris virgata*; LTC, litter total carbon; LTN, litter total Nitrogen; LTP, litter total phosphorus. Different capital letters indicate significant differences with degraded levels. Different lowercase letters indicate significant differences with litter types in the same degraded level (*P* < 0.05).
